# NETME: on-the-fly knowledge network construction from biomedical literature

**DOI:** 10.1007/s41109-021-00435-x

**Published:** 2022-01-06

**Authors:** Alessandro Muscolino, Antonio Di Maria, Rosaria Valentina Rapicavoli, Salvatore Alaimo, Lorenzo Bellomo, Fabrizio Billeci, Stefano Borzì, Paolo Ferragina, Alfredo Ferro, Alfredo Pulvirenti

**Affiliations:** 1 Department of Physics and Astronomy, University of Catania, Catania, Italy; 2 Department of Clinical and Experimental Medicine, University of Catania, Catania, Italy; 3 Department of Maths and Computer Science, University of Catania, Catania, Italy; 4 Department of Computer Science, University of Pisa, Pisa, Italy

**Keywords:** Network analysis, Knowledge graph, Text mining

## Abstract

**Background:**

The rapidly increasing biological literature is a key resource to automatically extract and gain knowledge concerning biological elements and their relations. Knowledge Networks are helpful tools in the context of biological knowledge discovery and modeling.

**Results:**

We introduce a novel system called *NETME*, which, starting from a set of full-texts obtained from *PubMed*, through an easy-to-use web interface, interactively extracts biological elements from ontological databases and then synthesizes a network inferring relations among such elements. The results clearly show that our tool is capable of inferring comprehensive and reliable biological networks.

**Supplementary Information:**

The online version contains supplementary material available at 10.1007/s41109-021-00435-x.

## Introduction

The increasing amount of scientific literature is posing new challenges for scientists. Identifying the most relevant articles dealing with a topic is not straightforward, leading to the high chance of missing essential references and relevant literature. In particular, in research areas like biology or bio-medicine, thanks to fast-track publication journals, the number of published papers increases significantly fast.

On the other hand, network analysis has become a critical enabling technology to understand mechanisms of life, living organisms, and in general, uncover the underlying fundamental biological processes. Examples of applications include: (i) analyzing disease networks for identifying disease-causing genes and pathways (Barabási et al. 2010); (ii) discovering the functional interdependence among molecular mechanisms through network inference and construction Szklarczyk et al. 2016; (iii) releasing Network-based inference models with application on drug re-purposing (Himmelstein et al. 2017).

In the last few years, thanks to the availability of sizeable open-access article repositories such as *PubMed* Central (Beck 2010), arxiv (https://arxiv.org) bioarxiv (https://www.biorxiv.org/) as well as ontology databases which hold entities and their relations (Lambrix et al. 2007), the research community has focused on text mining tools and machine learning algorithms to digest these corpora and extract valuable semantic knowledge from them. Text mining (Cohen 2005), and Natural Language Processing (Krallinger et al. 2005) tools employ information extraction methods to translate unstructured textual knowledge in a form that can be easily analyzed and used to build a functional network (i.e. a network in which the relations between two entities are not necessarily physical but can be indirect), or knowledge graphs (Szklarczyk et al. 2016; Dörpinghaus et al. 2019; Nicholson and Greene 2020). This technology allows us to infer putative relations among molecules, such as understanding how proteins interact with each other or determining which gene mutations are involved in a disease. In the context of biology and biomedicine, the Biological Expression Language (BEL) (Slater 2014), or Resource Description Framework (RDF) (McBride 2004) have been widely applied to convert a text in semantic triplets having the following form: <subject, predicate, object>. The subject and object represent biological elements, whereas the predicate represents a logical or physical relationship between them (Szklarczyk et al. 2016; Himmelstein and Baranzini 2015).

However, the implementation of biological text mining tools requires highly specialized skills in Natural Language Processing and Information Retrieval. Therefore, several ecosystems and tools have been implemented and made available to the bio-science community. Relevant tools include PubAnnotation (Kim et al. 2019), a public resource for sharing annotated biomedical texts based on the “Agile text mining” concept; PubTator (PTC) (Wei et al. 2019), a web service for viewing and retrieving bio-concept annotations (for genes/proteins, genetic variants, diseases, chemicals, species, and cell lines) in full-text biomedical articles. This latter tool annotates all *PubMed* abstracts and more than three million full texts. The annotations are downloadable in multiple formats (XML, JSON, and tab-delimited) through the online interface, a RESTful web service, and bulk FTP. Another interesting tool is SemRep (Rindflesch and Fiszman 2003), which extracts relationships from biomedical sentences in PubMed articles by mapping textual content to an ontology that represents its meaning. To establish the binding relation, SemRep relies on internal rules (called “indicator rules”), which map syntactic elements, such as verbs, prepositions, and nominalization, to predicates in the Semantic Network. We also mention Hetionet (Himmelstein et al. 2017), a heterogeneous network of biomedical knowledge that unifies data from a collection of several available databases and millions of publications. Also, the edges are extracted from omics-scale resources and consolidated through multiple studies or resources. Finally, in Yuan et al. (2019) authors propose a minimally supervised approach for knowledge-graph construction based on 24,687 unstructured biomedical abstracts. Authors included entity recognition, unsupervised entity and relation embedding, latent relation generation via clustering, relation refinement, and relation assignment to assign cluster-level labels. The proposed framework can extract 16,192 structured facts with high precision.

Starting from our previous work (Muscolino et al. 2021), we introduce *NETME* a novel web-based app (available at https://netme.click/ website, and https://github.com/alemuscolino/netme.git github repository), which is capable of extracting knowledge from a collection of full-text documents. The tool orchestrates two different technologies:

- A customized version of the entity-linker *TAGME* (Ferragina and Scaiella 2010) (called *OntoTAGME*) for extracting network nodes (i.e., genes, drugs, diseases) from a collection of full-text articles.
- A software module, developed on top of SpaCy (Honnibal et al. 2020) and NLTK (Loper and Bird 2002) libraries, that derives relations (edges) between pair of nodes. Edges are weighted according to their frequency within the collection of full-texts used to create the on-fly knowledge graph.

These inferred networks are handy in biomedicine, where it is essential to understand the difference between various components and mechanisms, such as genes and diseases, and their relations, such as up-regulation and binding. Therefore, the tool helps scientists fast identify reliable relations among the biological entities under investigation, based on their occurrences and mentions in *PubMed* ’s articles.

The novelties with respect our previous work (Muscolino et al. 2021) include:

- The sentence’s grammatical structure is extracted by Spacy linguistic annotations. Such a structure includes the word types (parts of speech) and how the words are related to each other. In the previous NETME release, the nltk bottom-up and top-down approach were employed for building the syntactic tree of each document sentence. Furthermore, the Spacy’s Matcher has been used to identify verbs’ passive forms. With this approach the system is now capable of properly establishing the correct edge direction.
- In Muscolino et al. (2021), the proposed system was able to build a network composed of only genes, diseases, and drugs. Now, thanks to the extension we made on *OntoTAGME*, our new system is able to build networks composed of much more biological entities such as: genes, variants, diseases, drugs, compounds, molecular function, biological proves, pathways, enzymes, etc.
- Finally, we designed and implemented a new module to handle the disambiguation among gene symbols and the acronyms of diseases or other biological elements. In fact, in many documents, the authors assign acronyms for very long biological elements that are usually equal to genes symbols.

To the authors’ knowledge, *NETME* is the first tool that allows to interactively synthesize biological knowledge-graphs on-the-fly starting from a *PubMed* query.

The paper is organized as follows. Section “The *NETME* model” introduces *NETME* system together with its components. Section “The annotation tool” provides the technical details of the back-end and the front-end of *NETME*. Section “Experimental analysis” reports two different case studies that allow evaluating *NETME* ’s prediction qualitatively. The first one is focused on: (i) recovering known gene interactions; (ii) avoid false-negative ones. For this purpose, we selected a subset of gene-gene interactions in KEGG/REACTOME (Kanehisa and Goto 2000; Kanehisa 2019, 2000; Fabregat et al. 2017) by making use of STRING API. More precisely, such interactions were obtained by selecting 100 random gene-gene interactions (manually curated in KEGG or REACTOME database) for each of the following STRING text-mining score intervals: 500-600,600-700, 700-800, 800-900, $>= 900$. Next, we selected the first 100 pairs of non-interacting genes from the Negatome 2.0 database (Blohm et al. 2013; Smialowski et al. 2009) in order to understand if *NETME* can avoid false-negative interactions. The experiment yielded accuracy values from 58% when the STRING text-minig score is in [500, 600] interval, to 84% when the value of such a score is higher than 900. Whereas, the second case study is focused on building a “CD147-genes” interaction network through selected papers containing valuable information about CD147 gene. We compared the network returned by *NETME* against a manually-curated network derived from these selected papers. The experiment yielded 98% sensitivity and 100% specificity. Therefore, both experiments clearly showed the high reliability of *NETME* inferred networks. Moreover, we have also assessed the *NETME* performance for inferring “CD147-diseases” interactions by selecting 100 random interactions from DisGenNET, and the same “abstracts” used by DisGenNET for inferring these interactions. *NETME* detected 63 True Positive values out of 100, revealing a sensitivity of 63% Sect. “Conclusion” ends the paper and sketches future research directions.

## The *NETME* model

A Knowledge Graph (also known as a semantic network) is a systematic way to connect information and data to knowledge. It represents a collection of interlinked descriptions of entities, real-world objects, and events, or abstract concepts, obtained from knowledge-bases such as ontologies $\left( O_1,O_2,\cdots , O_k\right)$. Basically, a semantic network is defined as a graph $G=(V,E)$ where entities are in *V*, and relationships in *E*. Each relation represents a connection between entities of one (intra-relationship) or more (inter-relationship) ontologies (Nettleton 2014). Therefore, there might exist a relation $e =(v_1,v_2) \in E$ where $v_1 \in O_i$ and $v_2 \in O_j$ with $i \ne j$.

An ontology is a formal description of knowledge as a set of domain-based concepts in relationships among them. As a result, the ontology does not only introduce a shareable and reusable knowledge representation, but it can also provide new knowledge about the considered domain (Xiaoke and Lin 2012).

*NETME* builds a biomedical knowledge graph starting from a set of *n* documents obtained through a query to the *PubMed* database. Papers can be sorted by relevance (default) or publication date. Users can also provide a list of PMCID/PMID or a set of PDF documents. The inferred network contains biological elements (i.e., genes, diseases, drugs, enzymes) as nodes and edges as possible relationships.

**Fig. 1:**
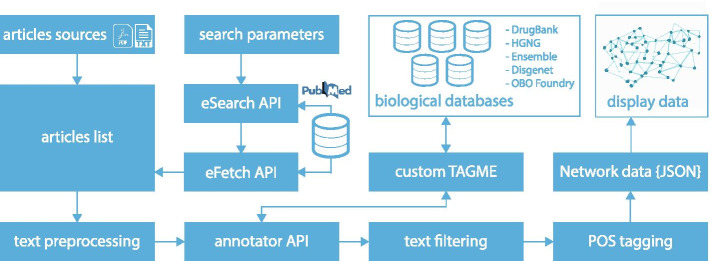
*NETME* pipeline architecture

In Fig. 1 we outline the architecture of *NETME*. The user provides the query terms to perform the search on *PubMed*, and she may directly provide PDFs or PMCIDs/PMIDs of other pertinent documents. Then *NETME* begins to create the network as follows:

1. First, *OntoTAGME* converts the full-text of the input documents into a list of entities (nodes) using literature databases and ontologies (such as GeneOntology Consortium 2004, Drug-Bank Wishart et al. 2017, DisGeNET Piñero et al. 2019, and Obofoundry Smith et al. 2007) as corpus. These entities will be the knowledge graph nodes. Note that, Obofoundry contains a several ontologies, but only the following have been currently used in our model: GO, DO, PW, BTO, PRO, AEO, PATO, CL and CLO.
2. Next, an NLP model based on Python SpaCy (Honnibal et al. 2020), and NLTK (Loper and Bird 2002) libraries, is executed to infer the relations among nodes entity-nodes belonging to the same sentence ($S_i$) or to the adjacent ones ($S_i$, $S_{i+1}$) of the same document. Such relationships indicate disease treatment, genes regulations, molecular functions, gene-gene interactions, gene-disease interactions, gene-drug interactions, drug-disease interactions, disease-disease interactions and drug-drug interactions.

The final network will contain both directed and undirected edges according to the predictions made by the model. At the end of the process, the network will be rendered through Cytoscape JS. The following two subsections provide the details of these two phases.

### *OntoTAGME*: Ontology oN Top Of *TAGME*

*TAGME* Ferragina and Scaiella (2010) is a state-of-the-art entity linker for annotating Wikipedia pages mentioned in an input text. The tool searches for sequences of words (spots) that can be linked to pertinent Wikipedia pages (entities) that explain those words in that context. The use of Wikipedia as corpus allows to enrich texts with explanatory links in order to provide a structured knowledge for any unstructured fragment of the text. These links are then used for drawing a network of relationships among the extracted spots.

To mitigate ambiguity and polysemy, *TAGME* computes a $\rho$ value $\in [0,1]$ for each Spot-Entity (Node) association, and keeps only those ones having the $\rho$ value higher than an established user threshold. This value estimates the “goodness” of the annotation compared to other possible associations in the input text. A suitable use of $\rho$ ensures the highest accordance among the extracted spots.

Due to the topics-generality of the Wikipedia corpus used by *TAGME*, several non-biological spots could be extracted during the annotation procedure. To overcome this limitation, we developed a customized version of *TAGME*, called *OntoTAGME*, which makes use of several ontology and literature databases, such as: GeneOntology (GO) (Consortium 2004), DiseaseOntology (DO) (Schriml et al. 2018), PathwayOntology (PW) (Petri et al. 2014), BRENDA tissueenzyme source (BTO) (Gremse et al. 2010), ProteinOntology(PRO) (Natale et al. 2016), Anatomical Entity Ontology (AEO) (Bard 2012), Phenotype And Trait Ontology (PATO) (http://obofoundry.org/ontology/pato.html), Cell Ontology (CL) (Diehl et al. 2016), Cell Line Ontology (CLO) (Sarntivijai et al. 2014), DrugBank (Wishart et al. 2017), Disgenet (Piñero et al. 2019), HGNC (Gray et al. 2016), ENSEMBL (Birney 2004), CIViC (Griffith et al. 2017), and PharmGKB (Whirl-Carrillo et al. 2012). The usage of topic-specific ontology databases ensures reduced disambiguation errors and therefore yields highly reliable knowledge graphs inference.

**Fig. 2:**
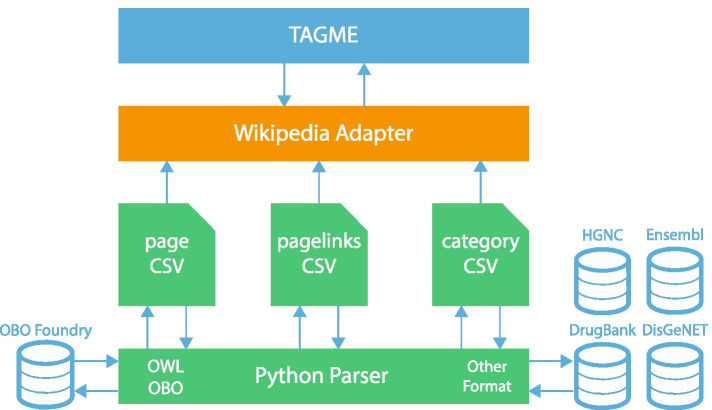
*OntoTAGME* pipeline architecture

The integration consisted of releasing a new intermediate python layer (Python Parser in Fig. 2), and a customized two-steps procedure (Wikipedia Adapter module in Fig. 2) for converting ontology databases in a *wikipedia-like* structure. The Python layer transforms a generic ontology or database in a list of CSV files: pages.csv, pageslink.csv and category.csv. The pages.csv stores the name of each biological element, and all possible synonyms. The pageslink.csv contains all the relationships among the nodes of the ontology. Finally, the category.csv has the type of each element extracted from the ontology or database entry (i.e Genes, Diseases, Drugs).

Next, a two-steps procedure is triggered to convert each row of the page.csv file into an XML file containing a unique ID generated by our system, the name (title), type (category) and the description (page’s body) of the considered biological element. Since an element *j* could have several linked pages “LPs” (i.e. DOID:0002116 is a DOID:10124), or redirected pages “RPs” due to synonyms (CD147 is a synonym of BSG), the process generates a tuple $\left<uniqueID_{j},\; uniqueID_{k}\right>$ for each element *k* belonging to LPs, and a tuple $\left<uniqueID_{j},\; uniqueID_{i}\right>$ for each element *i* belonging to RPs. These tuples are then stored in the SQL files “wiki-latest-pagelinks” and “wiki-latest-redirect”, respectively.

Finally, the SQL and XML files are used to generate the complete *OntoTAGME* network. It contains 331 thousand of main nodes, 700 thousand of synonyms, and 4 million of relationships.

#### Ontology databases

In order to build the *OntoTAGME* annotation networks we used the following nine ontology and six bio-databases.

DrugBank Wishart et al. (2017) contains data about drugs name, drugs synonyms, drug-drug interaction, and other comprehensive drug-target information. The database release used in our project is the v5.1 which contains 13, 367 drugs entries, including 2, 611 approved small molecule drugs, 1, 300 approved biotech (protein/peptide) drugs, 130 nutraceuticals and over 6, 315 experimental drugs. Additionally, 5, 155 non-redundant protein (i.e. drug target/enzyme/transporter/carrier) sequences are linked to these drug entries.

HGNG (HUGO Gene Nomenclature Committee) Gray et al. (2016) assigns unique and informative gene symbols and names to human genes. Standardized HGNC approved nomenclature is used in publications and biomedical databases to remove ambiguity and facilitate communication between researchers worldwide. The last database release contains more than 40, 000 approved gene symbols of which over 19, 000 are for protein-coding genes. The HGNC also names a set of small and long non-coding RNA genes and pseudo-genes (659 since 2017). The genes are grouped on the basis of several shared characteristics such as homology, associated phenotype and encoded protein function.

Ensembl Birney (2004) contains genome annotation (i.e genes, variation, regulation and comparative genomics) across the vertebrate sub-phylum and key model organisms. This tool is also able to compute multiple alignments, predicts regulatory function and collects disease data. The last complete version of the Ensembl database has been downloaded through their FTP service, and then integrated in *OntoTAGME* thanks to Python Parser layer. All data in Ensembl are used in combination with those coming from HGNC to detect Genes name and symbols within a text.

Disgenet Piñero et al. (2019) contains collections of genes and variants associated with human diseases. It integrates data from scientific literature, GWAS catalogues, expert curated repositories and animal models. Additionally, several original metrics are provided to assist the prioritization of genotype–phenotype relationships. DisGeNET releases two types of databases, Gene-Disease Associations and Variant-Gene Associations.

CIViC Griffith et al. (2017) is an expert-crowd-sourced knowledge-base for Clinical Interpretation of Variants in Cancer describing the therapeutic, prognostic, diagnostic and predisposing relevance of inherited and somatic variants of all types. CIViC is committed to open-source code, open-access content, public application programming interfaces (APIs) and provenance of supporting evidence to allow for the transparent creation of current and accurate variant interpretations for use in cancer precision medicine.

PharmGKB Whirl-Carrillo et al. (2012) is an interactive tool for researchers investigating how genetic variation affects drug response. It displays genotype, molecular, and clinical knowledge integrated into pathway representations and Very Important Pharmacogene (VIP) summaries with links to additional external resources. A user may search and browse the knowledge-base by genes, variants, drugs, diseases, and pathways through the website: http://www.pharmgkb.org).

OBO Foundry Smith et al. (2007) is the Open Biological and Biomedical Ontology (OBO) Foundry. It provides well-formed and scientifically accurate ontology thanks to the collaboration of ontology developers. They contribute to develop an evolving set of principles and common syntax based on ontology models that ensure the proper functioning of the system. In *NETME*, we use the following list of ontology:

- Gene Ontology (GO) Consortium (2004) project provides a uniform way to describe the functions of gene products from organisms across all kingdoms of life and thereby enable analysis of genomic data. it contains more than 44 thousand GO terms, 8 millions of annotations, 1.5 millions of gene products and nearly 5 thousand species.
- Human Disease Ontology (DO) Schriml et al. (2018) is a standardized ontology for human disease with the purpose of providing the biomedical community with consistent, reusable and sustainable descriptions of human disease terms, phenotype characteristics and related medical vocabulary disease.
- Pathway ontology (PW) Petri et al. (2014) is a controlled vocabulary for pathways that provides standard terms for the annotation of gene products.
- PRotein Ontology (PRO) Natale et al. (2016) defines taxon-specific and taxon-neutral protein-related entities in three major areas: proteins related by evolution; proteins produced from a given gene; and protein-containing complexes.
- BRENDA tissue / enzyme source (BTO) Gremse et al. (2010) is a structured controlled vocabulary for the source of an enzyme comprising tissues, cell lines, cell types and cell cultures.
- Anatomical Entity Ontology (AEO) Bard (2012) is an ontology of anatomical structures that expands CARO, the Common Anatomy Reference Ontology, to about 160 classes using the is_a relationship; it thus provides a detailed type classification for tissues. The AEO is useful in increasing the amount of knowledge in anatomy ontology, facilitating annotation and enabling interoperability across anatomy ontology.
- Phenotype And Trait Ontology (PATO) (http://obofoundry.org/ontology/pato.html) is used in conjunction with other ontologies such as GO or anatomical ontology to refer to phenotypes. Examples of qualities are red, ectopic, high temperature, fused, small, edematous and arrested.
- Cell Ontology (CL) Diehl et al. (2016) is designed as a structured controlled vocabulary for cell types. This ontology covers cell types from prokaryotes to mammals. However, it excludes plant cell types. One of the main uses of the CL is to describe samples used in transcriptomic and functional genomics studies, such as FANTOM5, ENCODE and LINCS.
- Cell Line Ontology (CLO) Sarntivijai et al. (2014) is a community-driven ontology that is developed to standardize and integrate cell line information and support computer-assisted reasoning.

The data relating to the number of nodes and relationships extracted from each mentioned ontology have been listed in Table 1

**Table 1:** Number of nodes and edges per ontology

Ontology name	Nodes number	Edges number
go	43917	142086
doid	10862	29938
pr	326811	846366
pw	2619	6210
cl	10809	34410
clo	44712	91966
aeo	248	523
bto	6515	9378
pato	4610	13027

### Network edge inference

Once the network nodes have been extracted the system will annotate their position and their main characteristics within the text. We capture the significant elements in each sentence, by making use of the parts of speech (POS tags). Then through a syntactic analysis we verify the coherence of the extracted elements. Indeed, sentences have an internal organization that can be represented using a tree. Solving a syntax analysis problem for a sentence consists of looking for predefined syntactic forms which, like a tree, branch out from the single words. The main syntactic form is the sentence (S) which contains noun phrases (NP) or verb phrases (VP) that are formed by further elementary syntactic forms such as nouns (N), verbs (V), determiners (DET), etc (see Table 3). All these information will be used by the textual analysis phase to infer relations between them.

A transition-based dependency parser is then used to first check the syntactic coherence and then build the syntactic tree. The dependency parser component inside the spaCy library jointly learns sentence segmentation and labelled dependency parsing. The parser uses a variant of the non-monotonic arc-eager transition-system (Honnibal and Johnson 2015), with the addition of a break transition to perform the sentence segmentation. Nivre’s (2005) pseudo-projective dependency transformation is also used to allow the parser to predict non-projective parses. The parser is trained through an imitation learning objective. It follows the actions predicted by the current weights and, at each state, it determines which actions are compatible with the optimal parse that could be reached from the current state. The weights are updated in a way that the scores assigned to the set of optimal actions is increased, while scores assigned to other actions are decreased. Note that more than one action may be optimal for a given state.

Once *OntoTAGME* have extracted the set of nodes $n_{1}, \ldots , n_{z}$ from a list of *N* full-text documents $\left[ p_{1}, p_{2}, \ldots , p_{N}\right]$, the edge inference module of *NETME* (developed on top of the Python library NLTK Loper and Bird 2002 and spaCy (Honnibal et al. 2020)) starts to establish any verbal relationships between those pairs of nodes. When two or more nodes are detected within a sentence or adjacent sentences, the syntactic analyzer extracts the parts of speech and syntactic dependencies within the sentence. For each sentence we then get a set of labelled tokens $lt_{1}, lt_{2} \ldots , lt_{k_i}$. Each token is a tuple of the following form $\{token,POS, dependency\_label\}$, where POS and Dependency label are valued with the data present in Table 3.

**Fig. 3:**
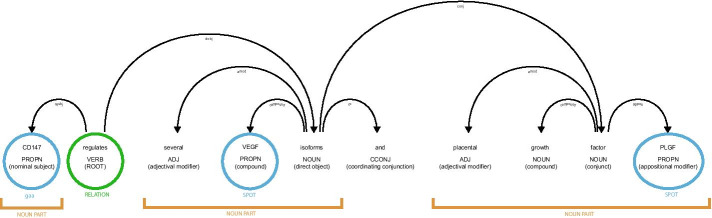
*NETME* example of POS extraction and coherence checking for the sentence *[...] CD147 regulates several VEGF isoforms and placental growth factor (PLGF), and it has unique effects on trophoblastic function.[...]*. Through *OntoTAGME* we detect the spots [*“BSG”, “VEGFA”, “PGF”*]. After the syntactic analysis, three noun parts are identified (the phrase spots, highlighted via orange segments): two of them (“VEGF” and “PLGF”) have a joint relationship with the first (“CD147”). The verbal part is the root between the two pairs of nouns (“CD147”—“VEGF”), (CD147—“PLGF”)

Irrelevant POS are filtered out (stop-words, URLs, etc.), we keep only the useful verb forms and the nodes which correspond to the noun parts. A final pruning phase is also executed in which we use: (i) POS tag labels and dependency labels to check if the syntactic link between the verb form and the annotations is correct and consistent, as described in the Fig. 3; (ii) a dictionary of biological verb forms to check if they are pertinent. The surviving nodes and verb forms will allow to generate network edges.

In our final network, each edge $e=\left( a,b\right)$ is weighted with three parameters: the term frequency and inverse document frequency (tf.idf), the medium relatedness (*mrho*) and the biological degree (bio). More specifically, tf.idf is a measure of how much information the edge provides, namely if it is common or rare across all input documents. In formula, we compute $\mathrm{tf.idf}(e,p,P) = \mathrm{tf}(e,p)* \mathrm{idf}(e,P)$.

**Fig. 4:**
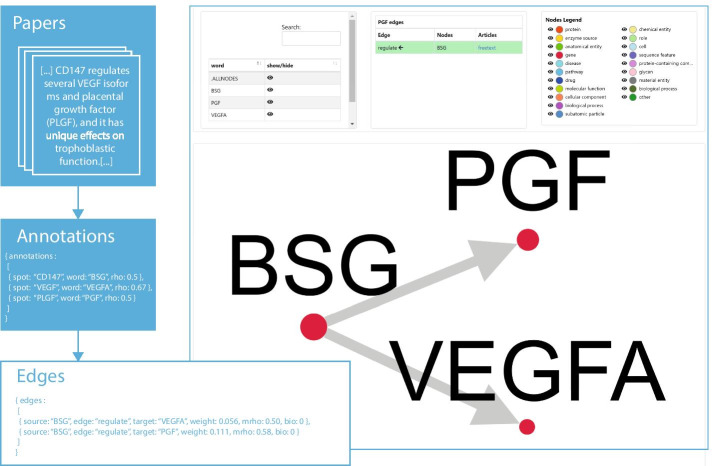
Example of annotation of the sentence *[...] CD147 regulates several VEGF isoforms and placental growth factor (PLGF), and it has unique effects on trophoblastic function.[...]*. Through *OntoTAGME* we detect the spots [*“BSG”, “VEGFA”, “PGF”*], and after the syntactic analysis and noise reduction steps, we detect two valid edges: [*“BSG”, “regulate”, “VEGFA”*] and [*“BSG”, “regulate”, “PGF”*]. Note that “regulate” is a biological verb forms and it has bio parameter set to 0

Where, term frequency $\mathrm{tf}(e,p)$ is the frequency of edge *e*, is defined as $\mathrm{tf}(e,p) = {f_{e,p}}/{\sum _{e'\in {p}}f_{e',p}}$, with ${f_{e,p}}$ representing the number of times that edge *e* occurs in paper *p*. The inverse document frequency $\mathrm{idf}(e,P)$ is a measure of how much information the edge *e* provides. It is defined as $\mathrm{idf}(e,P) = log {N}/{|\{p\in {P}:e\in {p}\}|}$, where *N* is the number of documents analyzed by the query such that $N=|P|$, and $|\{p\in {P}:e\in {p}\}|$ is the number of documents where the edge *e* appears. The parameter *mrho* measures the relatedness of the labels starting from the $\rho$ value assigned by *OntoTAGME* to the two annotations involved, i.e. $mrho(e) = \frac{\rho _{a}*\rho _{b}}{2}$. The *bio*-parameter is the cosine similarity (having a value ranging from 0 to 1) between the inferred relationship and a set of biological verb forms (see Table 2). Figure 4 provides an example of such an annotation.

**Table 2:** List of biological verb forms

Verb forms		
Activate	Downregulate	Reduce
Affect	Enhance	Regulate
Associates	Express	Release
Block	Find	Reveal
Cause	Inactivate	Stimulate
Contain	Increase	Trigger
Control	Induce	Ubiquitination
Decrease	Interacts	Upregulates
Detect	Overexpress	
Display	Produce	

**Table 3:** List of POS tag and syntactic dependency labels

POS tag		Dependency label	
Symbol	Meaning	Symbol	Meaning
ADD	email	acl	clausal modifier of noun (adjectival clause)
AFX	affix	acomp	adjectival complement
CC	conjunction, coordinating	advcl	adverbial clause modifier
CD	cardinal number	advmod	adverbial modifier
DT	determiner	agent	agent
EX	existential there	amod	adjectival modifier
FW	foreign word	appos	appositional modifier
HYPH	punctuation mark, hyphen	attr	attribute
IN	conjunction, subordinating or preposition	aux	auxiliary
JJ	adjective	auxpass	auxiliary (passive)
JJR	adjective, comparative	case	case marking
JJS	adjective, superlative	cc	coordinating conjunction
LS	list item marker	ccomp	clausal complement
MD	verb, modal auxiliary	compound	compound
NFP	superfluous punctuation	conj	conjunct
NN	noun, singular or mass	csubj	clausal subject
NNP	noun, proper singular	csubjpass	clausal subject (passive)
NNPS	noun, proper plural	dative	dative
NNS	noun, plural	dep	unclassified dependent
PDT	predeterminer	det	determiner
POS	possessive ending	dobj	direct object
PRP	pronoun, personal	expl	expletive
PRP$	pronoun, possessive	intj	interjection
RB	adverb	mark	marker
RBR	adverb, comparative	meta	meta modifier
RBS	adverb, superlative	neg	negation modifier
RP	adverb, particle	nmod	modifier of nominal
SYM	symbol	npadvmod	noun phrase as adverbial modifier
TO	infinitival “to”	nsubj	nominal subject
UH	interjection	nsubjpass	nominal subject (passive)
VB	verb, base form	nummod	numeric modifier
VBD	verb, past tense	oprd	object predicate
VBG	verb, gerund or present participle	parataxis	parataxis
VBN	verb, past participle	pcomp	complement of preposition
VBP	verb, non-3rd person singular present	pobj	object of preposition
VBZ	verb, 3rd person singular present	poss	possession modifier
WDT	wh-determiner	preconj	pre-correlative conjunction
WP	wh-pronoun, personal	predet	None
WP$	wh-pronoun, possessive	prep	prepositional modifier
WRB	wh-adverb	prt	particle
		punct	punctuation
		quantmod	modifier of quantifier
		relcl	relative clause modifier
		xcomp	open clausal complement

## The annotation tool

*NETME* is provided with a front-end developed in PHP and Javascript, in which the network rendering is performed through the CytoscapeJS library (Franz et al. 2015). Its back-end, which integrates *OntoTAGME*, is written in Java and communicates with both Python NLTK (Loper and Bird 2002) and SpaCy (Honnibal et al. 2020) libraries for the NLP module. *PubMed* search is performed with the Entrez Programming Utilities (https://www.ncbi.nlm.nih.gov/books/NBK25501/), a set of server-side programs providing a stable interface to the Entrez database and to the query system at the National Center for Biotechnology Information (NCBI).

**Fig. 5:**
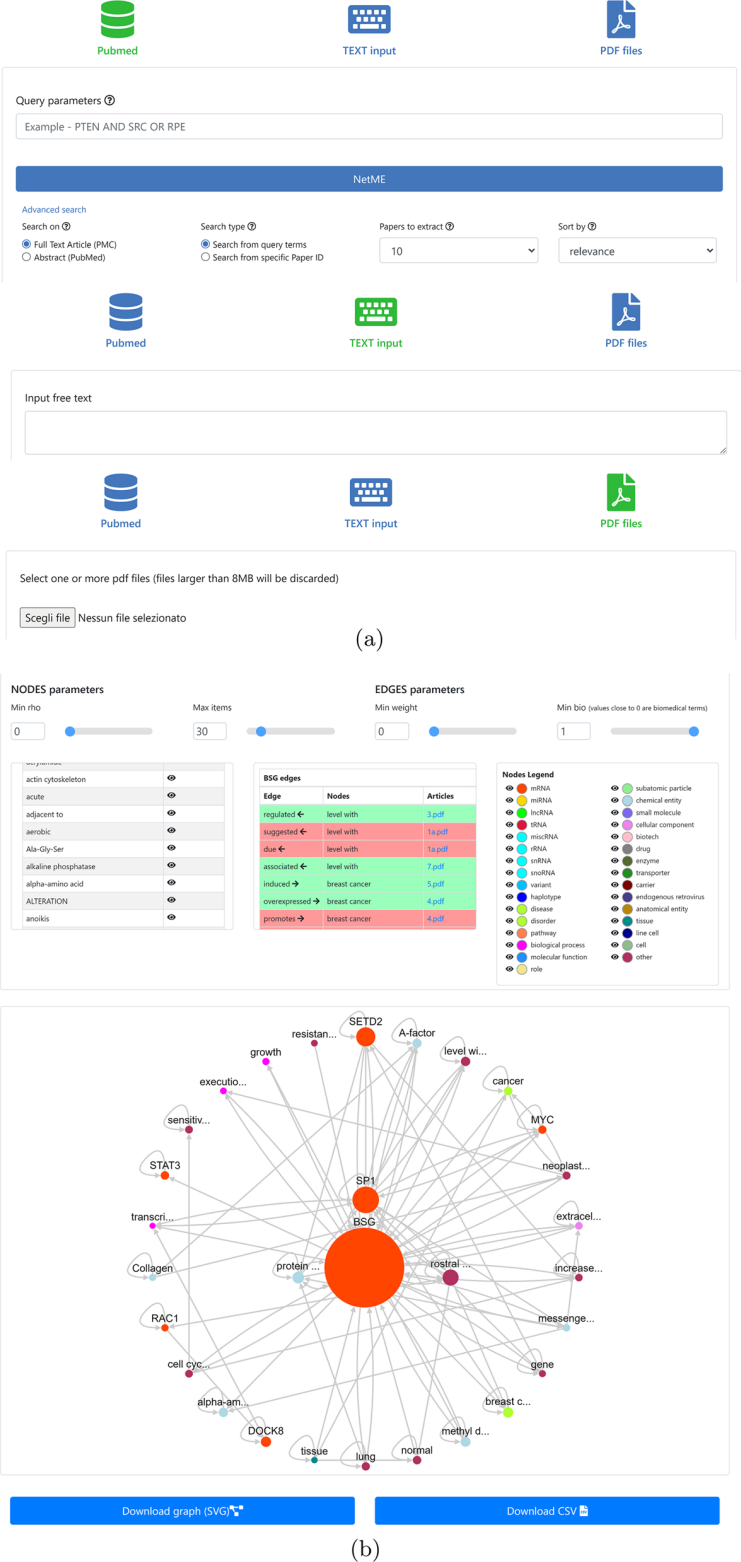
*NETME* web interface in (**a**), generated network in (**b**)

**Fig. 6:**
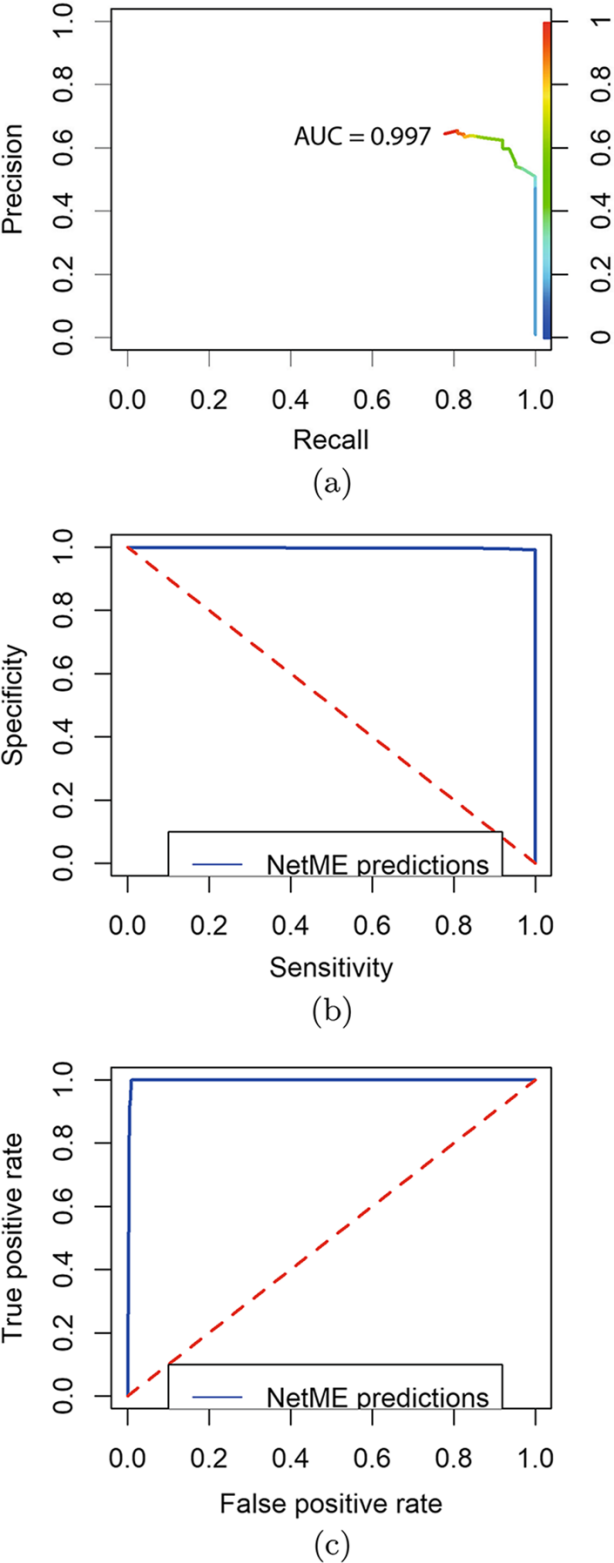
Metrics of BSG-network performed by *NETME*. The plots show **a** Precision/Recall curve; **b** Sensitivity/Specificity; **c** True positive rate/False Positive Rate. The red dashed line in **b**, **c**, indicates the expected result if the used method was random that is any method which, given a pair of nodes, elects whether between them there is a link with a probability of 0.5

**Fig. 7:**
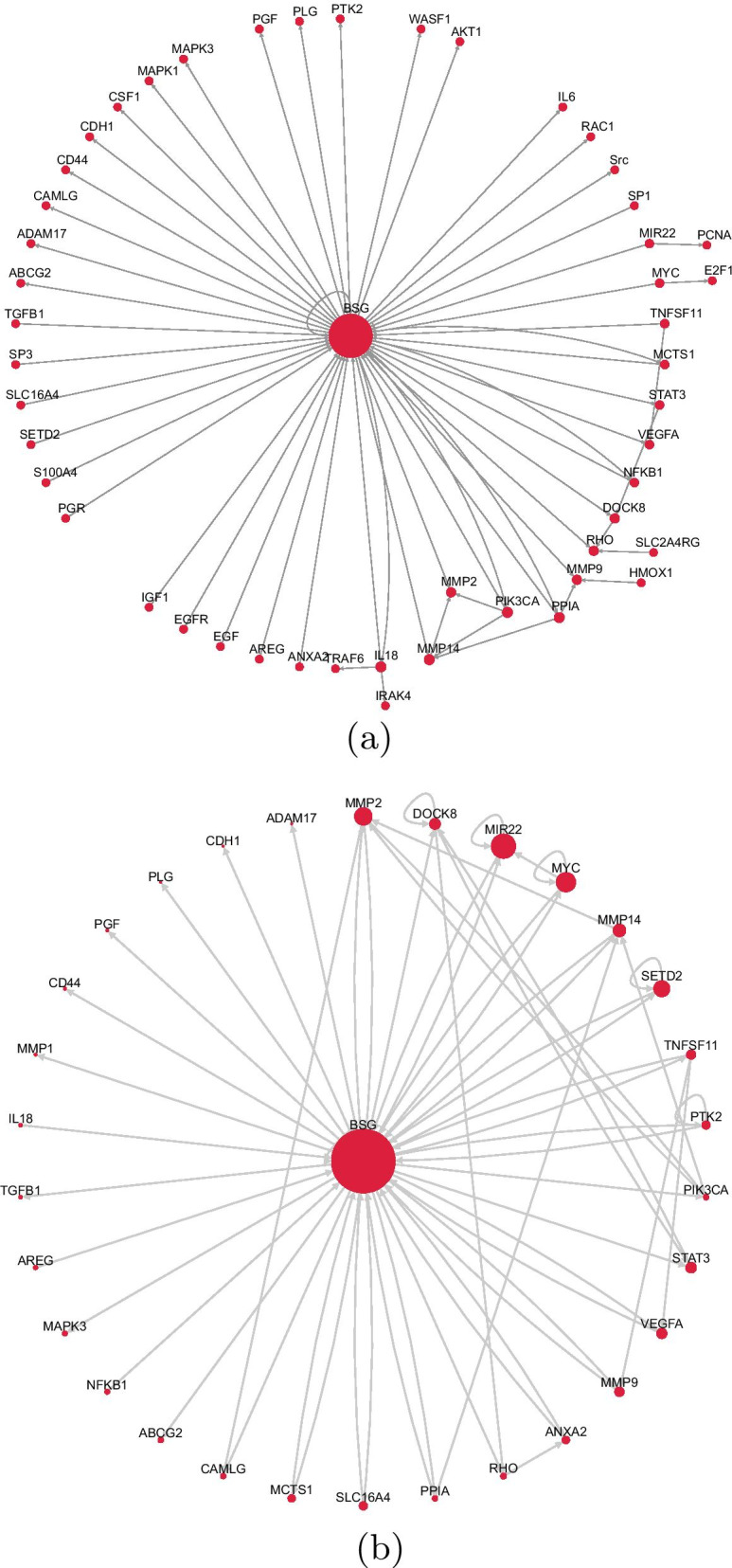
**a** Depicts the pathway constructed by hand from the selected papers (Jiang et al. 2014; Kong et al. 2014; Ke et al. 2012; Grass and Toole 2016; Xiong et al. 2014; Rucci et al. 2010; Ding et al. 2017; Ulrich and Pillat 2020; Wang et al. 2014; Kong et al. 2014; Kirk et al. 2000), with CD147(BSG) as the central node. **b** Shows the molecular mechanisms summarised in the knowledge network developed by *NETME* in accordance with the same papers used in **a** *NETME* shows that CD147 is a potent inducer of metalloproteinases (MMPs) such as MMP2, MMP14 and MMP9 as reported in Xiong et al. (2014); Rucci et al. (2010); Ding et al. (2017). Furthermore, the overexpression of CD147, which results in increased phosphorylation of PI3K(PIK3CA), Akt(AKT1), leads to the secretion of vascular endothelial growth factor (VEGFA) in several biological contexts such as KSHV infection Xiong et al. (2014); Rucci et al. (2010). In addition to its ability to induce MMPs, CD147 regulates spermatogenesis, lymphocyte reactivity and MCT system, in particular MCT1 and MCT4 (MCTS1 and SLC16A4) expression (Xiong et al. 2014; Kirk et al. 2000). Our results also show that CD147 can increase the expression of ATP-binding cassette transporter G2 (ABCG2) protein, regulating its function as a drug transporter, as mentioned by Xiong et al. for MCF-7 cells (Xiong et al. 2014). *NETME* identifies also BSG as an upstream activator of STAT3, highlighting its involvement in tumor development in agreement with the literature (Wang et al. 2014). As summarized by our knowledge network, CD147 is regulated by various inflammatory mediators, such as RANKL (TNFSF11), denoting its involvement in inflammatory processes (Grass and Toole 2016; Rucci et al. 2010). Among the potential activators of BSG, *NETME* also find the transcription factor c-Myc (MYC) (Kong et al. 2014)

*NETME* is equipped with an easy-to-use web interface providing three major functions (see Fig. 5): (i) Pubmed query-based network annotation; (ii) user-provided free-text network annotation; (iii) user-provided PDF documents network annotation.

In the *query-based network annotation*, the user provides a list of keywords, which are employed to run a query on *PubMed*, or a list of article ids. The top resulting papers are retrieved and then the network inference procedure is run. Several parameters can be set by the user (or left with default values) such as: the number of top article to retrieve from *PubMed*, and the criteria used to sort papers (relevance or date).

In the *user-provided free-text network annotation*, users provide a free text which is then input to the network inference procedure.

In the *user-provided PDF documents network annotation*, users give a set of PDF documents which are then input to the network inference procedure.

The result of the network inference procedure is a direct graph (network) which shows all inference details in three main tables containing: the list of extracted papers, the list of annotations, and the list of edges together with their weight.

The user can then click on a node of the network to view all incoming and outgoing connections, or she can click on an edge to display its type and the verbal relation between the nodes it connects.

## Experimental analysis

To analyze the reliability of *NETME*  knowledge graphs, we performed two case studies. The first one aims at providing a comprehensive analysis of *NETME*  performance by checking its ability to predict known relations between genes drawn from Kyoto Encyclopedia of Genes and Genomes - KEGG (Kanehisa and Goto 2000; Kanehisa 2019, 2000) or REACTOME (Croft et al. 2010; Joshi-Tope 2004; Croft et al. 2013) pathways and, on the other hand, its ability to avoid inferring false connections between proteins by using the Negatome 2.0 database (Blohm et al. 2013; Smialowski et al. 2009). The second case study is more specific and focuses on building a network based on some selected publications that contain valuable information specific to the CD147 gene. Such a network is then compared against a manually-curated one derived from the same papers by a bio-expert. In both cases, the performance of *NETME* has been measured in terms of a precision/recall curve.

### Case study 1

The first case study focuses on assessing *NETME*  performance through its capability to recover known gene interactions. For this purpose, we selected a subset of gene-gene interactions from KEGG/REACTOME by making use of STRING API. More precisely, such interactions were obtained by selecting 100 random gene-gene interactions for each of the following STRING text-mining score intervals: 500–600, 600–700, 700–800, 800–900, $\ge 900$ (listed in Additional files 1, 2, 3, 4, 5, respectively). These interactions form the true-positive set.

Next, we selected 100 random pairs of non-interacting genes from the Negatome 2.0 database as a true-negative set (listed in Table 5). For each interacting gene-pairs, we queried *NETME* with the papers used by STRING to infer the interactions. On the other hand, to annotate non-interacting genes, we queried *NETME* with the pair of genes of interest, selecting the top 20 papers from *PubMed*. Accuracy, sensitivity, specificity and PPV values, detected by *NETME*, are listed in Table 4 The results clearly show that NETME produces reliable results when the annotations are performed on top of relevant literature (STRING text-mining score higher than 700). On the other hand, when the STRING text-mining score is lower than 700, the NETME performances degrade in accordance with STRING predicted confidence as highlighted by their score . The reason behind such a behaviour is due: (i) not enough literature about these interactions; (ii) the interactions have been inferred by human curators as a combination of other interactions occurring in the text. Furthermore, when the text-mining score is small, STRING predictions could be wrong. In fact, as reported in Szklarczyk et al. (2016), a score of 500 would indicate that roughly every second term of an interaction might be erroneous (i.e., a false positive). Therefore, the computed value of accuracy, sensitivity, specificity and PPV could be incorrect.

**Table 4:** Metrics on *NETME* ’s ability to predict known interactions (from KEGG/Reactome) and non-interactions (from Negatome 2.0) between genes

Text-mining score interval	Accuracy (%)	Sensitivity (%)	Specificity (%)	PPV (%)
500–600	58.5	31	86	68.8
600–700	66.5	47	86	77.05
700–800	72.5	59	86	80.8
800–900	73.5	61	86	81.3
$\ge 900$	84	82	86	85.4

**Table 5:** List of the first 100 pairs of non-interacting genes from the Negatome 2.0 database.The column “SOURCE” indicates the starting gene, instead the column “TARGET” indicates the gene to which the action of the source gene is directed

Non-interacting genes from Negatome 2.0			
SOURCE	TARGET	SOURCE	TARGET
AKT1	TSC1	MAD2L2	MAD1L1
ARAF	BCL2L1	NCK1	EGFR
ARAF	BCL2	OSM	LIFR
BCL10	BIRC3	PARD3	LIMK1
BCL2L1	MAVS	PDGFC	FLT1
BMPR1A	TGFB1	PFN4	ACTB
BMPR1A	BMP5	PGF	KDR
BMPR1A	BMP6	PIAS3	STAT1
BMPR1B	TGFB1	PIK3CG	PIK3R2
BMPR1B	BMP5	PKN1	RPS6KA1
BMPR1B	BMP6	PKN1	RPS6KA3
BMPR2	BMP2	PKN1	MAP3K2
CCND1	MCM2	PKN2	RPS6KA1
CCR3	CCL3	PKN2	RPS6KA3
CCR3	CCL4L2	PKN2	MAP3K3
CD274	CD28	RB1	SMAD3
CD274	CTLA4	RBL2	SMAD3
CD274	ICOS	RIPK1	TNFRSF10A
CD3G	ZAP70	RIPK1	TNFRSF10B
CD74	NOTCH1	SFN	TSC1
CDKN1B	TSC1	SH3KBP1	TNFRSF14
CSF2	IL3RA	SMAD1	ANAPC10
CTNNB1	HSP90AA1	SMAD4	ANAPC10
CTNNB1	DDIT3	SOCS3	JAK2
CTNND1	IL2	STIM1	TRPC6
CTNND1	APC	TANK	RBCK1
CTNND1	CTNNA1	TBC1D7	TSC2
CTNND1	CTNND1	TFDP1	CDK2
CTNND1	CTNNB1	TFDP1	CCNA1
DKK1	WNT1	TICAM1	TLR4
DKK1	SOST	TJAP1	F11R
DVL1	TSC1	TJAP1	CLDN1
EIF3I	ACVR2A	TJAP1	TJP1
EIF3I	ACVR1	TNF	EGFR
EIF3I	TGFBR1	TRADD	TNFRSF10A
EP300	CD44	TRADD	TNFRSF10B
ERBB2	PIK3R2	TRAF6	IRF3
ETS1	CREBBP	TSC1	CDKN1B
FOXO1	TSC1	VAV1	SHC1
GRAP2	SOS1	VEGFB	KDR
GRAP2	CBL	VEGFB	FLT4
HDAC2	RELA	VEGFC	FLT1
HIPK2	MDM2	VIPR2	RAMP1
HSPA4	BAX	VIPR2	RAMP2
IGF2	IGF1R	VIPR2	RAMP3
IL15	IL2RA	VWF	F8
IL1A	EGFR	YWHAB	TSC1
IL22	IL10RA	YWHAE	TSC1
IL4R	IL13	YWHAZ	TSC1
KDR	FLT1	NFKBIA	CREB3L2

### Case study 2

Many tools (Alaimo et al. 2020) and computational models rely on existing network databases, such as KEGG (Kanehisa and Goto 2000; Kanehisa 2019, 2000) and Reactome (Croft et al. 2010; Joshi-Tope 2004; Croft et al. 2013). However, despite the enormous amount of available data, these databases are still incomplete and therefore have partial information (Menche et al. 2015). As an example, KEGG includes approximately one-third of the known genes. In this case study, we have chosen CD147, also known as Basigin (BSG) or EMMPRIN, as a starting point for the gene-gene interactions network construction. This gene represents an example of a biological element that should be supplemented to the KEGG network since it is not currently described in their pathways. Among the bibliography consulted to build the network manually, we have carefully selected 11 papers containing a significant amount of helpful information for our purpose. On the other hand, in this case study, we have also assessed the capabilities of *NETME* in inferring CD147-diseases relations. For this purpose we selected 100 random interactions from DisGenNET (Piñero et al. 2019), as well as the same abstracts used by DisGenNET for inferring such interactions (listed in Additional file 6).

CD147 is a transmembrane glycoprotein of the immunoglobulin superfamily, expressed in many tissues and cells, which is known to participate in several high biological and clinical relevance processes and is a crucial molecule in the pathogenesis of several human diseases (Xiong et al. 2014). Recently Wang et al. (2020) discovered an interaction between host cell receptor CD147 and SARS-CoV-2 spike protein, together with Angiotensin-Converting Enzyme 2 (ACE2), as an entry point for SARS-CoV-2.

In this direction, CD147 is an example of how a missing crucial gene within a biological network can compromise scientists’ efforts to understand certain molecular phenomena. In literature, there are many valuable tools (Himmelstein et al. 2017; Himmelstein and Baranzini 2015) to integrate the missing information into bio-databases, such as KEGG. However, the most reliable approach in terms of accuracy and updated information remains the manual curation of such networks through careful and time-consuming literature analysis. On the other hand, a manually constructed network provides partial information due to the limited number of articles that a scientist could read. Our second case study affords this issue by providing a practical example of how *NETME* can create valuable networks by analyzing quickly and automatically larger sets of publications. The set of 11 selected papers, described in Fig. 7a, was analyzed by a bio-expert to derive a CD147-genes interactions network manually. This process resulted in 50 genes and 64 interactions, as shown in Fig. 7a. Next, by using the same set of papers, we run *NETME* with no upstream filter. The automatically generated network consisted of 86 genes and 139 relationships between them (see Fig. 7a, b). As the manually curated network consists of genes and proteins, only elements from these two categories were selected for the evaluation. This was performed by considering edges with the lowest “bio” score for each node pair. Qualitatively, this network includes most of the interconnections mentioned in the papers, thus providing a reliable and comprehensive overview of the molecular function of Basigin. Quantitatively, *NETME* achieved an accuracy of 98.99%, a sensitivity of 100%, a specificity of 98.98%, and a positive predicted value of 46.32%.

Figure 6a–c depicts the precision/recall curve (AUC 0.997), the sensitivity/specificity curve and the True positive rate/False Positive Rate one. The construction of the curves considered all possible gene-pairs and their edges.

Finally, we queried *NETME* with the selected 100 random CD147-diseases interactions in DisGenNET, selecting the same PubMed abstract used by DisGenNET for inferring those interactions. *NETME* detected 63 True Positive values out of 100, revealing a sensitivity of 63%

It is essential to stress that *NETME*  allows us to extract a satisfactory and valid amount of information in a few minutes, compared to a manual search that may take days or weeks. We also believe that this case study is significant because, in the evaluation, we considered not only the presence of a link between two nodes but even more closely the type of edge, hence the adequacy and specificity of the annotated edge in its biological context.

## Conclusions

In this paper, we have introduced *NETME* system to infer on-the-fly knowledge-graphs from a collection of either full-text papers obtained from *PubMed* or user-provided ones. It has been implemented upon a customized version of *TAGME*, called *OntoTAGME*, in connection to a syntactic analysis module developed on top of the Python NLTK and SpaCy libraries. Our results clearly show that *NETME* allows extracting reliable knowledge graphs in a few minutes or hours compared to a manual search that could take several days or weeks. The completeness of the extracted knowledge increases when the documents used by *NETME* comprehensively describe the desired topic under study. To evaluate *NETME*, we performed two case studies. The first one tested the ability of *NETME* in recovering relationships between genes. The experiment yielded accuracy ranging from 58%, when using low reliable relations (i.e. False Positives) from STRING, to 84% when such STRING relations are very reliable. At the same time, the second case study tested the ability of *NETME* in integrating knowledge about genes starting from a selected set of papers. The experiment yielded 98% sensitivity and 100% specificity. Therefore, both experiments clearly showed the high reliability of *NETME* ’s inferred networks.

Future work will include: (i) the construction of knowledge-graphs from all the open-access papers stored in PubMed Central; (ii) the integration of all Obofoundry ontology within *OntoTAGME*; (iii) the design of a more effective algorithm to select the pertinent papers on which *NETME* has to be applied (Ponza et al. 2019, 2020); and finally, add a methodology that allows to extract context-based relationships

## Supplementary Information

**Additional file 1**. The json files storing all gene1-gene2 pairs used in the first case study having String scores ranging from 500 to 600. The main key of each record is the name ofthe two genes concatenated by "-". The lists of documents, are under the sub-keys "PMID" and "PMC".

**Additional file 2**. The json files storing all gene1-gene2 pairs used in the first case study having String scores ranging from 600 to 700. The main key of each record is the name ofthe two genes concatenated by "-". The lists of documents, are under the sub-keys "PMID" and "PMC".

**Additional file 3**. The json files storing all gene1-gene2 pairs used in the first case study having String scores ranging from 700 to 800. The main key of each record is the name ofthe two genes concatenated by "-". The lists of documents, are under the sub-keys "PMID" and "PMC".

**Additional file 4**. The json files storing all gene1-gene2 pairs used in the first case study having String scores ranging from 800 to 900. The main key of each record is the name ofthe two genes concatenated by "-". The lists of documents, are under the sub-keys "PMID" and "PMC".

**Additional file 5**. The json files storing all gene1-gene2 pairs used in the first case study having String scores greater than 900. The main key of each record is the name ofthe two genes concatenated by "-". The lists of documents, are under the sub-keys "PMID" and "PMC".

**Additional file 6**. The json files storing all BSG-Disease available in DisGenNET. The lists of documents, are under the sub-keys "PMCID".

## Data Availability

The datasets generated and analysed during the current study are available at the following URL https://netme.click/. Additional files for reproducibility purpose are provided as supplementary materials.

## Abbreviations

BEL: Biological Expression Language
RDF: Resource Description Frame-work
PTC: Pub-Tator
spots: Words
HGNC: HUGO Gene Nomenclature Committee
APIs: Application programming inter-faces
VIP: Very Important Pharmacogene
GO: Gene Ontology
DO: Human Disease Ontology
PW: Pathway ontology
PRO: PRotein Ontology
BTO: BRENDA tissue/enzyme source
AEO: Anatomical Entity Ontology
PATO: Phenotype And Trait Ontology
CL: Cell Ontology
CLO: Cell Line Ontology
POS: Parts of speech
S: Sentences
NP: Noun phrase
VP: Verb phrase
N: Nouns
V: Verbs
DET: Determiners
mrho: Medium relatedness
BSG: Basigin
ACE2: Angiotensin-Converting Enzyme 2
PLGF: Placental growth factor
MMPs: Metalloproteinases
VEGFA: Vascular endothelial growth factor
ABCG2: ATP-binding cassette transporter G2
MYC: Transcription factor c-Myc
